# Peroneal muscle response to single-leg drop-jump and unexpected leg-drop in young and middle-aged adults before and after one session of neuromuscular training

**DOI:** 10.1186/s11556-023-00321-8

**Published:** 2023-06-17

**Authors:** Roee Hayek, Uri Gottlieb, Itai Gutman, Shmuel Springer

**Affiliations:** Faculty of Health Sciences, Department of Physical Therapy, The Neuromuscular & Human Performance Laboratory, Ariel University, Medicine and Health Science Building, Rm 30, 40700 Ariel, Israel

**Keywords:** Postural control, Balance, Middle age, Aging

## Abstract

**Background:**

Changes in neuromuscular ability in middle age (MA) may lead to deterioration of postural control. The aim of this study was to investigate the anticipatory response of the peroneus longus muscle (PL) to landing after a single-leg drop-jump (SLDJ), and its postural response after an unexpected leg-drop in MA and young adults. A second aim was to investigate the influence of neuromuscular training on PL postural responses in both age groups.

**Methods:**

Twenty-six healthy MA (55.3 ± 4 years) and 26 healthy young adults (26.3 ± 3.6 years) participated in the study. Assessments were performed before (T0) and after (T1) PL EMG biofeedback (BF) neuromuscular training. Subjects performed SLDJ, and PL EMG activity in preparation for landing (% of flight time) was calculated. To measure PL time to activation onset and time to peak activation in response to an unexpected leg-drop, subjects stood on a customized trapdoor device that produced a sudden 30° ankle inversion.

**Results:**

Before training, the MA group showed significantly shorter PL activity in preparation for landing compared to the young adults (25.0% vs. 30.0%, *p* = 0.016), while after training there was no difference between the groups (28.0% vs. 29.0%, *p* = 0.387). There were no differences between groups in peroneal activity after the unexpected leg-drop before and after training.

**Conclusions:**

Our results suggest that automatic anticipatory peroneal postural responses are decreased at MA, whereas reflexive postural responses appear to be intact in this age group. A short PL EMG-BF neuromuscular training may have an immediate positive effect on PL muscle activity at MA. This should encourage the development of specific interventions to ensure better postural control in this group.

**Trial registration:**

ClinicalTrials.gov NCT05006547.

**Supplementary Information:**

The online version contains supplementary material available at 10.1186/s11556-023-00321-8.

## Introduction

In middle age (MA, 45–65 years), neuromuscular ability may be affected by age-related changes in the nervous system, muscle mass, strength, and flexibility, which may lead to a decline in various functions [1, 2]. Evidence suggests that impairments in balance and locomotion are widespread in MA. Up to 10–20% of people in this age group report having difficulty with mobility, despite having only mild and common health problems such as back pain [3–5]. Recognition of functional deterioration at MA may promote appropriate interventions that can impact performance in the third age [6, 7]. However, while many studies have demonstrated significant differences in function between young and old adults, few studies have focused on MA.

Postural control ability depends on intact function and integration of the visual, vestibular, somatosensory, and neuromuscular systems [8]. The performance of these systems, as well as their integration by the central nervous system, may decline with age [9]. Therefore, assessment of postural control during demanding tasks may be beneficial to detect early balance deterioration in MA.

Ankle muscles activity for postural control, termed the “ankle strategy,” is critical for maintaining balance [10]. While large ankle muscles such as the tibialis anterior, soleus, and gastrocnemius are essential for anterior–posterior stability [11], the peroneus longus muscle (PL) plays an important role in maintaining mediolateral stability [11–13]. The PL muscle prevents the ankle from involuntarily inversion during heel strike [12, 14], and has been found to activate ipsilaterally during inward perturbations, such as a push to the contralateral side [15]. PL activity after perturbation is also considered a primary reflex, that leads to additional responses to maintain mediolateral stability [15]. In a number of studies, a reduction in mediolateral instability has been associated with aging [16–18] and it has also been found to be related to falls [19]. Terrier and Reynard [19] found a significant association between age and mediolateral instability, and reported that such deterioration may begin as early as age 50 years. Therefore, it is important to further study the PL function in the middle-aged population.

Postural control strategies consist of voluntary responses, and automatic anticipatory and compensatory responses, that are faster than voluntary responses. The onset of automatic postural responses occurs with longer latencies compared with spinal stretch reflexes, which can activate the PL muscle during unexpected inversions [20]. Anticipatory activity of PL to maintain balance is distinct from activity in response to inversion perturbation. Therefore, to evaluate PL for balance control, its activity should be tested in different tasks. There is not much data on PL activity for postural control during aging; however, assessment of motor strategies during challenging tasks, such as landing, in MA may help to detect early deterioration of motor control [21–23]. In a previous study, we reported reduced stabilization performance after a single-leg drop jump in middle-aged adults compared with young adults [24]. Schmidt et al. [25] tested the activation onset of the PL after an unexpected inversion perturbation and found that the activation onset time increased significantly with age. Yet, there was no specific analysis of PL performance in MA, and automatic anticipatory responses were not tested.

Neuromuscular training has been shown to improve the speed and intensity of the balance response, even brief interventions, involving only one session have been shown to have acute effects on balance [26, 27]. Weight-bearing training has been shown to be more effective in improving PL activity, yet non-weight-bearing training may also have a beneficial effect [28]. Non-weight-bearing neuromuscular training may be less fatiguing and demanding [29, 30]. In addition, weight-bearing training may activate large muscle groups rather than a specific muscle [31], whereas open kinematic exercise may allow training a particular muscle [31, 32]. Visual feedback during exercise can increase the effectiveness and specificity of neuromuscular training [33]. One such method is feedback on muscle activity with electromyography biofeedback (EMG-BF) [33]. Visual biofeedback allows the trainer to learn how to control the contraction of a target muscle by displaying the activity of the muscle on a screen [34]. Therefore, a single session of open kinematic chain with visual biofeedback training can have an acute positive effect on PL activity.

To better understand mediolateral dynamic postural control ability in MA, we tested the response of the PL muscle after an unexpected leg-drop and its anticipatory response to landing after a single-leg drop-jump (SLDJ) in MA and young adults. Since training of the sensorimotor system is thought to influence the type or speed of response to stability disturbance [20], our second objective was to examine the influence of neuromuscular training on PL postural responses in both age groups. We hypothesized that before neuromuscular training, PL activation onset after an unexpected leg-drop would not differ between groups and that PL time to peak activation would be longer in MA compared with young adults. This hypothesis was based on a previous study that compared middle-aged and young adults and demonstrated differences in tibialis anterior time to peak after perturbation, whereas no differences were found in the activation onset time [35]. In addition, we expected that PL activity in preparation for landing (% of flight time) would be shorter in MA. Anticipatory muscle activity before landing after a jump is planned and organized by the central nervous system, which may be affected by aging [36, 37]. We also thought that neuromuscular training would improve PL postural response in both groups.

## Methods

### Participants

Twenty-six healthy MA individuals (aged 50 to 65 years) and twenty-six healthy young adults (aged 20 to 35 years) participated in the study. Participants were included if they were able to perform moderate to vigorous physical activity in daily living and leisure time. Exclusion criteria were musculoskeletal, neurological, vestibular, visual impairments, or other conditions that could significantly limit mobility and balance. In addition, subjects with even mild ankle injuries were not included because this could affect their PL activity [38]. Participants were recruited through public advertisements in the community and social media. All participants provided written informed consent before the study began. The study was approved by the Ariel University Ethics Committee (AU-HEA-SS -20,210,304).

### Procedure

Measurements were performed in a single session at the Neuromuscular and Human Performance Laboratory at Ariel University. At baseline, background variables were collected, including anthropometric data, physical activity level (using the International Physical Activity Questionnaire [IPAQ]) [39], leg dominance (by asking with which leg the participant would kick a ball) [40], and maximal grip strength using a handgrip dynamometer (Jamar®, 5030J1, Patterson Medical, Warrenville, IL, USA). Maximal grip strength was tested as a background variable because it has been previously reported to be associated with functional limitations at MA [7]. In addition, ankle eversion maximal voluntary contraction ([MVC], Newton [N]), was measured with a handheld dynamometer (HHD) (Lafayette Instrument, Manual Muscle Tester) attached to a custom-constructed testing environment, which is shown in Additional file 1: Appendix I. This testing environment avoided movement above the ankle and ensured that the HHD was placed on the lateral side of the foot, 2 cm proximal to the head of the fifth metatarsal, at a location marked by the examiner. PL EMG activity (root mean square [RMS] and median frequency [MDF]) was also recorded during MVC testing. Three measurements were taken for grip force and ankle eversion MVC, with 60 s rest between contractions. The maximal force result of each measurement was divided by the participant’s body mass to normalize the data and was used for data analysis. Then, subjects were assessed in a randomized order during SLDJ and unexpected leg-drop. The ankle eversion MVC tests and the SLDJ and unexpected leg-drop assessments were performed before (T0) and after (T1) neuromuscular training of the PL with EMG-BF.

**Fig. 1:**
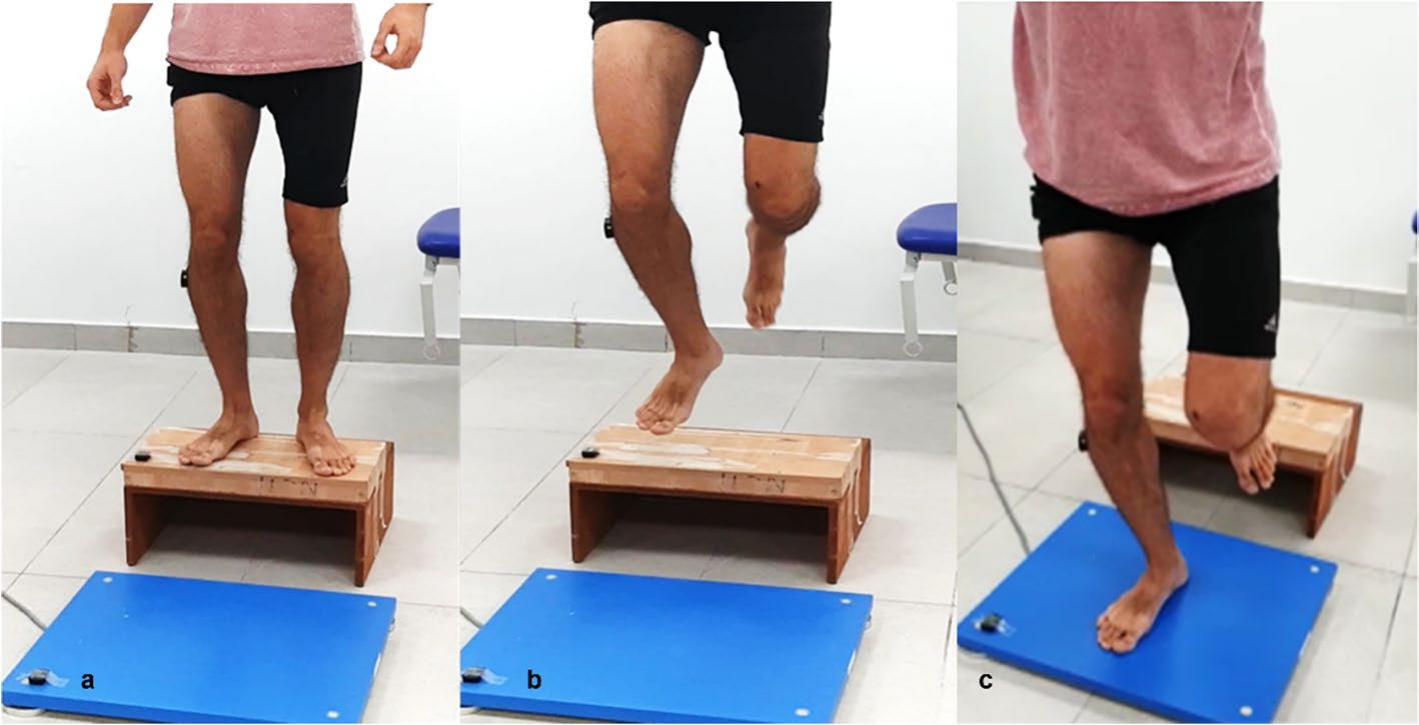
The single-leg drop-jump test. **a** Subject standing on 25 cm box, 5 cm behind the force plate. **b** The subject performs a single leg drop jump. **c** The subject is trying to stabilize on his dominant leg after landing on the force plate

For the SLDJ assessment, subjects jump from a 25-cm-high box onto a 50⨯60⨯5-cm force plate (Kistler, Switzerland). The starting position was barefoot with both feet on the box. Participants were then asked to jump off the box, land on the dominant leg in the center of the force plate, and stabilize as quickly as possible. The SLDJ test is shown in Fig. 1. No instructions were given for the position of the upper extremities. If the other leg touched the force plate or the participant failed to stabilize, the measurement was disqualified and counted as a failed jump. After each jump, subjects had a rest period of 30 s until a total of five successful trials had been collected. Prior to data collection, there was a visual demonstration and participants were asked to perform three practice jumps to ensure they understood the procedure.

**Fig. 2:**
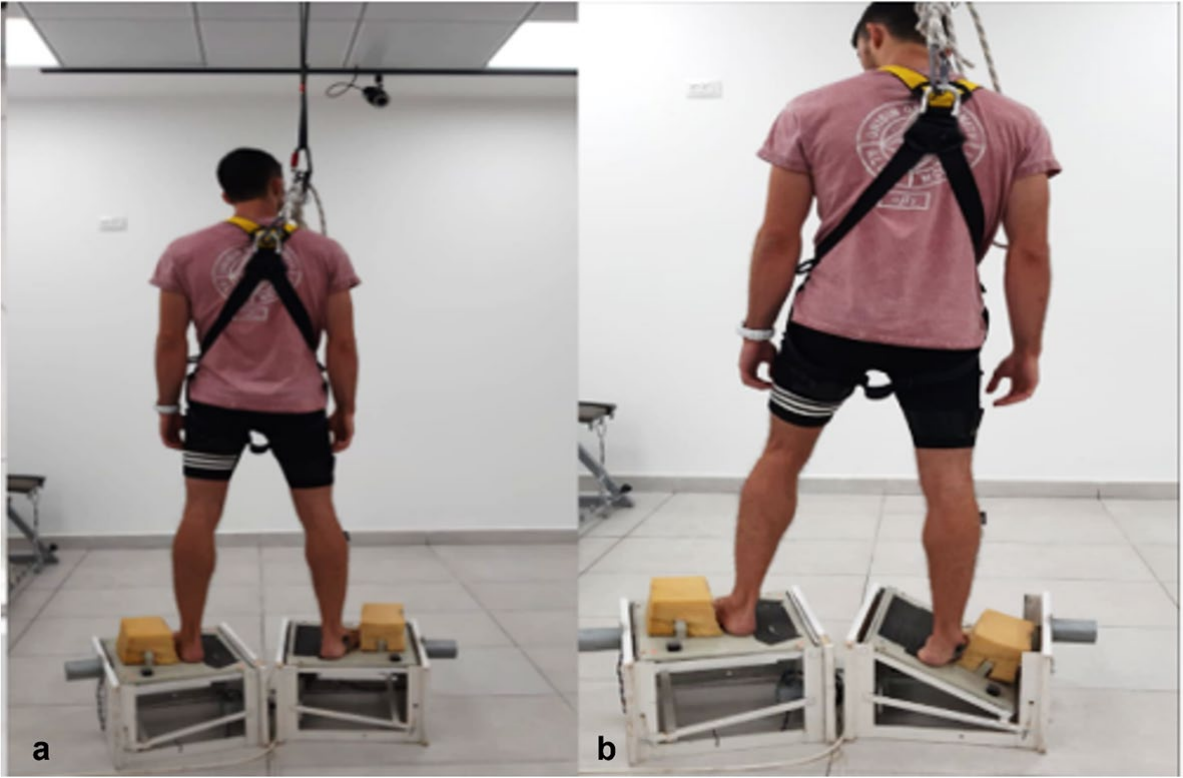
Peroneal muscle’s electrical activity reaction time to the unexpected leg-drop (perturbation). **a** Subject standing with his back to the examiner, on the moveable platform, before perturbation, secured by safety harness. **b** The subject standing with 30° right foot inversion, after perturbation

The unexpected leg-drop was studied using two platforms that allowed a sudden drop of 30° toward inversion at each leg, as described by Konradsen and Ravn [21]. Subjects stood on the platforms (each leg on a different platform) with their backs to the investigator. They were instructed to stand quietly, and the investigator made sure that they maintained this posture. The platforms on which the subjects stood were controlled remotely by the investigator. The investigator sat in front of the computer screen that monitored the subject’s PL EMG activity. After verifying that the muscle was in a quiet state, the investigator pressed a button, which triggered a drop on one of the two platforms (onset of perturbation). To ensure the safety of the subjects, they were secured with a safety harness attached to the ceiling. The unexpected leg-drop is shown in Fig. 2. Prior to data collection, the task was demonstrated verbally and visually, and participants had two practice trials for familiarization. Ten trials were recorded, five for each leg, in randomized order presented only to investigator. Perturbations were performed on both legs to reduce subject expectancy as much as possible. Data were analyzed for the dominant leg only.

To measure PL EMG activity, a Trigno™ Wireless EMG sensor (Delsys Inc., Boston, MA, USA) was placed over the PL muscle belly (“PL EMG”) according to Surface Electromyography for the Non-Invasive Assessment of Muscles (SENIAM) guidelines [41]. The area where the sensor was placed was cleaned with alcohol and shaved to reduce skin resistance. A wireless inertial motion unit (IMU) (Delsys Inc., Boston, MA, USA) was attached to the subject’s heel (“IMU heel”) 1 cm distal to the lateral malleolus, and an additional IMU was attached to the force plate (“IMU force plate”) to record jump onset (the moment the subject’s feet have left the box during the SLDJ) and landing time (touchdown) along with data received from the force plate. Two IMU’s were attached to the tilt platforms (“IMU rt/lt platform”) to detect the onset time of the perturbation during the unexpected leg-drop. The locations of the wireless EMG and IMU sensors are shown in Additional file 1: Appendix II.

### EMG-BF neuromuscular training

PL EMG-BF training was performed with the Trigno® Wireless Biofeedback System (Delsys Inc, USA) and the “PL EMG”, which allowed the acquisition of signals in real-time. Subjects were seated in a highchair and held their legs in the air to allow an eversion movement. A screen in front of the subjects displayed the EMG signal. During the exercise, subjects were asked to use their PL EMG signal to trace a parabola curve displayed on the screen. This simulated daily muscle activation with gradual contraction and relaxation [42]. The peak of the parabola was set at 65% of the maximal electrical activity measured before training, which is similar to the PL activity during walking [12], making the training demanding but not too strenuous. The neuromuscular training lasted five minutes, divided into rounds of eight seconds of muscle contraction and fifteen seconds of rest. Before the training began, the task was demonstrated, and the investigator made sure that the subjects understood the task. The PL EMG-BF is shown in Additional file 1: Appendix III.

### Data analysis

We used MATLAB (R2021a) to process PL RMS and MDF during ankle eversion MVC. Signals were filtered using a fourth-order Butterworth bandpass filter with a cutoff frequency of 20 Hz to 400 Hz.

PL EMG activity in preparation for landing (% of flight time) was calculated to represent the anticipatory response to landing during the SLDJ. The “PL EMG” was used to record muscle activity. The onset of the jump was defined as the root of the sum of the squares of the acceleration (ACC_XYZ) in the “IMU heel”. The “IMU force plate” and the data obtained from the force plate determined the landing time. PL activity prior to landing was divided by flight time to represent the percentage of PL activity in preparation for landing. See Additional file 1: Appendix IV for a further description of the calculation of PL activity in preparation for landing.

PL activation onset time and time to peak activation were calculated to represent the reflexive response to the perturbation during the unexpected leg-drop. The “PL EMG” was used to record muscle activity. The “IMU rt/lt platform” detected the side and exact onset of the perturbation. PL activation onset time to and time to peak activation were measured in relation to the onset of the perturbation. PL EMG time to activation onset was calculated as the time difference between the perturbation and the activation onset of PL activity. The EMG signals were filtered with a fourth-order Butterworth bandpass filter between 20 and 400 Hz and rectified using a root-mean-squared algorithm with a moving window of 25 ms.

The perturbation was identified by the first peak of the “IMU rt/lt platform” gyroscope signal. The gyroscope signal was filtered with a first-order bandpass Butterworth filter between 0.001 Hz and 5 Hz. Absolute maximal gyroscope signal was used to detect the falling side. The perturbation was identified as the beginning of the first peak of the gyroscope signal. The mean and standard deviation (SD) of the EMG RMS signal during the resting phase was calculated. The resting phase was defined as the 250 ms before the perturbation. The onset of PL activation was indicated when the EMG signal first raised above the mean + 5 SD of the quiet phase. This threshold (i.e.,5 SD) was chosen as suggested by Knight and Weimar [35]. The peak EMG amplitude was defined as the maximum RMS value of the EMG signal; therefore, the PL time to peak activation was defined as the time difference between the perturbation and the peak EMG amplitude. The calculation of PL activation onset time and time to peak activation was performed with a custom Python script.

### Statistical analysis

Normal distribution was assessed using the Shapiro–Wilk test revealed that all data were normally distributed except for the IPAQ. For descriptive statistics, quantitative variables were described with mean ± standard deviation (SD) or median and interquartile range (IQR), depending on the type of distribution. Categorical variables were summarized with frequencies and percentages. Demographic characteristics were compared between groups using the t test for normally distributed variables or the Mann–Whitney U test for nonnormally distributed variables. Demographic variables were considered as covariates if at least a moderate correlation (r > 0.3) was found with the PL activity during fall outcomes (i.e., PL activity in preparation for landing/ PL activation onset time and time to peak activation). Pearson or Spearman correlations were calculated as appropriate. Because no significant correlations were found, no covariates were included in the final analysis. The effect of age and EMG-BF training on PL EMG activity in preparation for landing was tested using repeated measures ANOVA and Bonferroni post hoc analysis.

Pearson correlation revealed a significant moderate correlation between PL activation onset time and time to peak activation (*r* = 0.3, *p* = 0.003). Consequently, multivariate analysis of variance (MANOVA) for repeated measures was performed to evaluate the effects of age and EMG-BF training on unexpected leg-drop performance (PL activation onset time and time to peak activation).

To determine fatigue after neuromuscular training, ankle eversion MVC, PL RMS, and MDF were compared before and after training in each group separately using a paired t test. Statistical analysis was performed using IBM SPSS Statistics, version 27.0. (Armonk, NY: IBM Corp), and significance was set at p < 0.05.

## Results

### Demographic and Background outcomes

Participants’ demographic and background variables are shown in Table 1. No significant differences were found between groups, except for age and grip strength normalized to body mass (0.51 in the MA group vs 0.61 in the young group, *p* = 0.012).

**Table 1:** Participants’ demographic characteristics

	Young (*n* = 26)	Middle-aged (*n* = 26)	*p*-value
**Age (years)**	26.3 ± 3.60	55.3 ± 4.01	** < 0.001**
**Gender: Female (%)**	13 (50%)	13 (50%)	1
**Leg dominance: right (%)**	26 (100%)	26 (100%)	1
**Height (m)**	1.68 ± 0.11	1.71 ± 0.09	0.431
**Weight (kg)**	67.3 ± 14.01	73.8 ± 14.6	0.121
**BMI (kg/m** ^2^ **)**	23.37 ± 2.51	25.0 ± 3.50	0.063
**Grip strength** ^a^ ** (KgF/Kg)**	0.61 ± 0.15	0.51 ± 0.13	**0.012**
**Ankle eversion MVC at T0** ^a^ ** (N/Kg)**	2.14 ± 0.61	2.30 ± 0.75	0.579
**Total physical activity (MET-min/week)**	3159 (8065–1905)	2592 (4898–1339)	0.241
**IPAQ time spent sitting (min/week)**	338.08 ± 124.31	356.15 ± 192.85	0.747

Mean ± standard deviation

Median (interquartile range)

*BMI*, Body mass index, *MVC* Maximal voluntary contraction, *IPAQ* International physical activity questionnaire, *METS* metabolic equivalent units, *METS-min* Metabolic equivalent of task-minuets

^a^Normalized to body mass

#### Single-leg drop-jump (SLDJ)

**Fig. 3:**
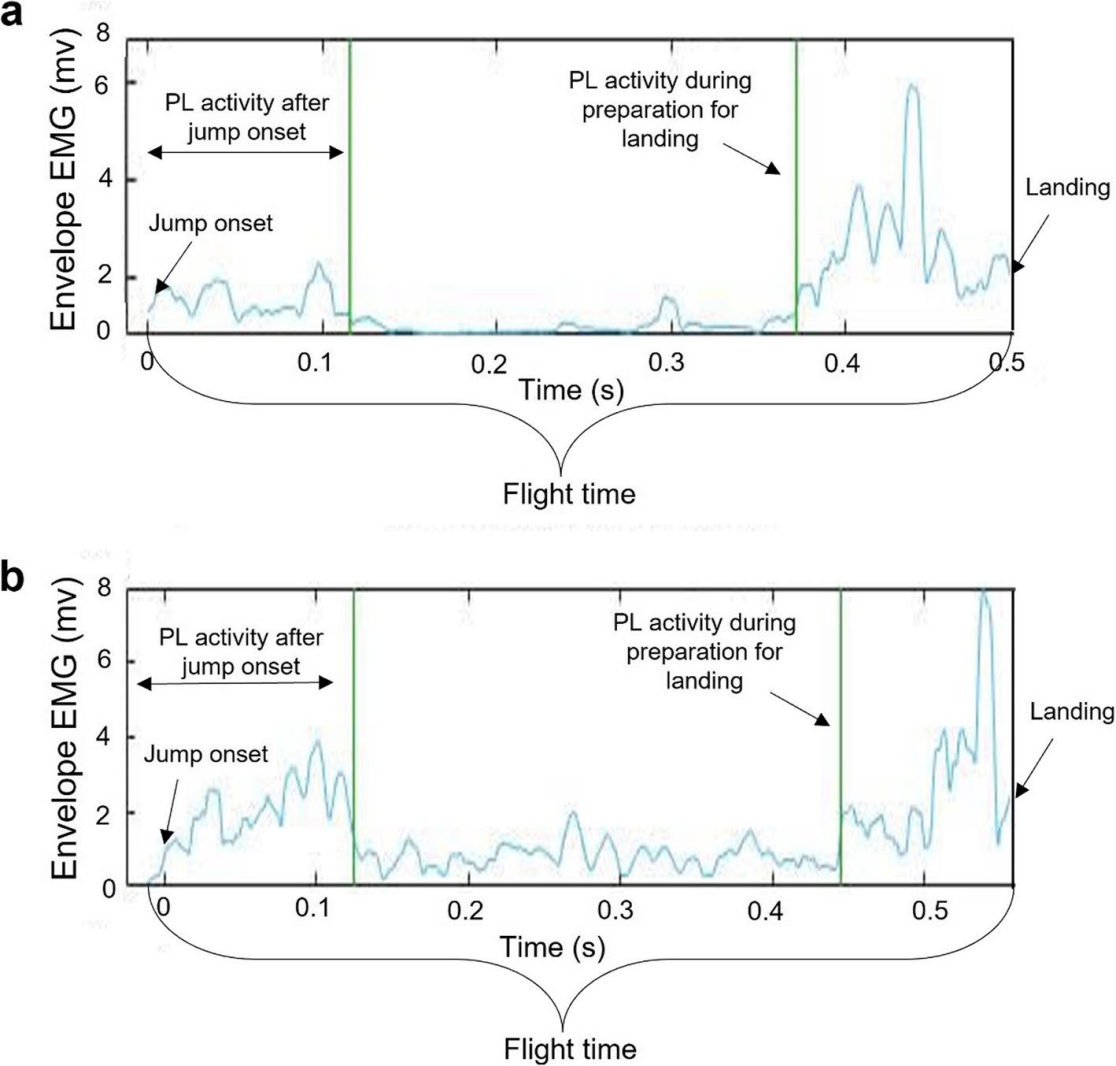
Peroneal EMG activity in preparation for landing during the SLDJ (before training), an example of one subject from each group. **a** Young adult subject aged 24 years; **b** Middle-aged subject aged 59 years

There was no significant effect for age (*p* = 0.092) or EMG-BF training (*p* = 0.201) on PL activity in preparation for landing. However, there was a significant interaction effect between age and EMG-BF training (*p* = 0.026) on PL activity in preparation for landing. PL Activity in preparation for landing (% of flight time) in both groups before and after training is shown in Table 2. Figure 3 presents an example of PL EMG activity in preparation for landing during the SLDJ (before training) from one subject in each group.

**Table 2:** Percentage of PL activity in preparation for landing

		Young (*n* = 25)	Middle-aged (*n* = 26)	*p*-value	Partial Eta squared
Percentage of PL activity in preparation for landing (% of flight time)	T0	30.02 ± 7.70	24.96 ± 5.31	**0.016**	0.113
	T1	28.98 ± 7.11	27.91 ± 7.73	0.387	0.015

Mean ± standard deviation

*PL* Peroneus longus, *EMG* Electromyography

Post-hoc analysis revealed that at T0, PL preparation was longer in the young group (30.0%, 95% CI: 26.7–33.4%) than in the MA group (25.0%, 95% CI: 22.8–27.1%) (*p* = 0.016, partial eta squared (η_p_^2^) = 0.113). At T1, no difference was found between groups in PL preparation (*p* = 0.387).

In the MA group, a significant difference was found in PL preparation between T0 and T1 (*p* = 0.013), while in the young group there was no difference between T0 and T1 (*p* = 0.484).

There were no differences in the number of jump failures between the MA and the young group at T0 (22 vs 15, *p* = 0.641) and at T1 (17 vs 12, *p* = 0.141).

#### Unexpected leg-drop

**Fig. 4:**
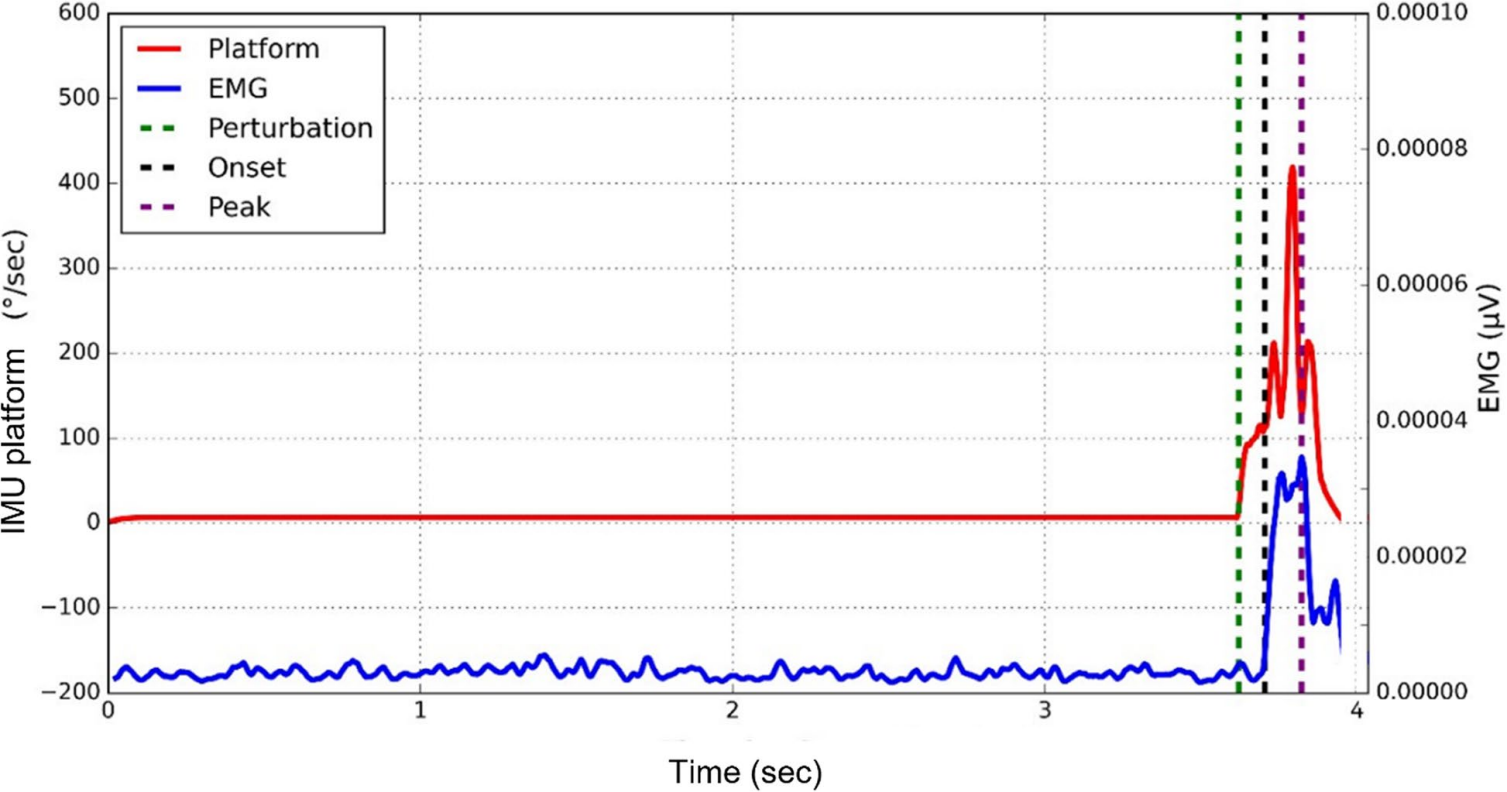
Peroneal EMG activation onset time and time to peak activation during the unexpected leg-drop. Blue line: PL EMG; Red line: IMU platform (gyroscope signal); Dashed black line: PL activation onset time; Dashed green line: Perturbation onset; Dashed purple line: PL time to peak activation

There was no significant effect for age (*p* = 0.082), EMG-BF training (*p* = 0.664), or interaction (age X EMG-BF training, *p* = 0.571) on PL outcomes during the unexpected leg-drop. PL time to activation onset and time to peak activation at T0 and T1 in both groups are presented in Table 3. Figure 4 shows an example of PL EMG activation onset time and time to peak activation during the unexpected leg-drop.

**Table 3:** Peroneal time to activation onset and time to peak activation during the unexpected leg-drop

		Young (*n* = 26)	Middle-age (*n* = 26)	*p*-value
Time to activation onset (ms)	T0	68 ± 10.01 (63–72)	74 ± 10.02 (68–80)	0.070
	T1	67 ± 10.13 (65–73)	75 ± 10.11 (69–81)	0.075
Time to peak activation (ms)	T0	149 ± 10.03 (137–162)	169 ± 10.02 (152–186)	0.069
	T1	151 ± 10.02 (138–166)	163 ± 10.01 (149–176)	0.230

Mean ± standard deviation (95% confidence interval)

#### The effect of neuromuscular training on PL fatigue

There was no change in ankle eversion MVC, and in PL RMS and MDF before and after the neuromuscular training, in both age groups, indicating no muscle fatigue post-training. The results of these outcomes are shown in Table 4.

**Table 4:** The effect of the neuromuscular training on peroneal fatigue

		T0	T1	*p*-value
Young (*n* = 26)	**Ankle eversion MVC (N/Kg)**	2.120 ± 0.51	2.151 ± 0.53	0.071
	**PL RMS (µV)**	0.115 ± 0.06	0.104 ± 0.05	0.120
	**PL MDF (Hz)**	95.612 ± 20.70	98.691 ± 18.51	0.269
Middle-age (*n* = 26)	**Ankle eversion MVC (N/Kg)**	2.250 ± 0.80	2.320 ± 0.71	0.141
	**PL RMS (µV)**	0.055 ± 0.04	0.054 ± 0.03	0.841
	**PL MDF (Hz)**	94.939 ± 20.81	97.550 ± 16.98	0.260

*MVC* Maximal voluntary contraction, *MDF* Median frequency, *RMS* Root mean square

## Discussion

This study tested the response of the PL muscle to SLDJ and unexpected leg-drop in young and MA adults. Our results suggest a decrease in PL anticipatory response for lading after a SLDJ in MA compared with young adults. Recent studies have shown that falls are common in MA [4, 43, 44], and have emphasized the role of an adequate lateral ankle roll mechanism in maintaining balance [15, 45, 46]. Our findings are of importance because there are few studies that have examined balance in the MA population.

Adequate anticipatory postural responses are important for maintaining dynamic stability [47]. A decrease in anticipatory responses correlates with poor mobility, deterioration of balance, and risk of injury [22, 48]. Peroneal muscles have been shown to be more active during preparation for landing than during walking or jogging [49]. Therefore, a reduction in the anticipatory response during this activity could have a greater impact on function. Although the SLDJ tested in the laboratory is not identical to everyday activities, it may resemble some activities, such as getting off a bus or train, as well as movements during hiking and sports that are commonly performed at MA. Therefore, the feedforward components of motor control in the jump landing strategy may well represent the anticipatory postural skills needed in many daily activities.

To our knowledge, the only comparison of PL anticipatory response to landing after SLDJ was performed in subjects with functional ankle instability, who were also found to have a decrease in PL anticipatory preparation for landing compared with healthy controls [22]. Although we found a significant difference in PL anticipatory response between MA and young adults, the clinical significance of this difference (i.e., mean difference of five percent) should be further investigated.

In contrast to PL anticipatory response when preparing to land after a jump, PL time to activation onset and time to peak activation after the unexpected perturbation were similar in both groups. Most of the available data examined the response of various lower extremity muscles to external perturbations in older and young adults [50, 51], but not in MA. To our knowledge, only Schmidt et al. [25] investigated age-related differences in PL time to activation onset after an inversion perturbation and, in contrast to our results, found a significant increase in PL time to activation onset at ages older than 50 years. Several explanations may be suggested for the different findings. Schmidt et al. [25] studied 120 healthy subjects and analyzed their PL response in relation to four age groups (A: 18–25 years, B: 26–35 years, C: 36–50 years, D: 51–70 years). None of the studied groups exclusively represented MA. In addition, 85 (71%) of the subjects were younger than 30 years; and although a regression analysis between age and peroneal reaction time was significant, it was not clear whether the average PL activation onset time of 65 ms in ages 26–35 groups (*n* = 15) was significantly shorter than the 71 ms measured in ages 51–70 (*n* = 10). These results are not significantly different from our results, which showed a non-significantly shorter latency of PL activation onset of 68 ms in the young group compared with 74 ms in the MA. Furthermore, we used a threshold of 5 SD to determine PL activation onset after the perturbation, whereas Schmidt et al. [25] used a lower threshold of 2 SD. Thus, further studies are needed to determine the effect of MA on PL time to activation onset and time to peak activation after unexpected perturbations. Moreover, the difference in PL anticipatory response at landing found between MA and young adults highlights the importance of testing both activities.

We found no significant difference between the age groups in PL time to peak activation after the inversion perturbation. In contrast, evaluation of tibialis anterior time to peak activation in MA and young adults performed by De Freitas et al. [35] showed a significant increase from the fifth decade. In addition, previous studies have found an increase in time to peak activation of various ankle muscles (with the exception of PL, which has not been studied) in the elderly compared with young adults. A longer time to peak activation of a muscle correlates with a lower rate of force generation and a reduction in sufficient postural responses [50, 52]. Possible reasons for the difference in results are the muscles tested, because different muscles may be affected differently, and the type, direction, or speed of the perturbation performed in each study, which may also affect muscle response [53]. Therefore, further studies are needed to better understand the changes associated with MA in time to peak activation of the ankle muscle.

Our results suggest that the automatic anticipatory postural responses of the ankle decrease at MA, whereas the reflexive postural responses seem to be intact in this age group. Whereas automatic postural responses require central nervous system control and may already be impaired at MA [8, 54], reflexive responses depend primarily on nerve conduction velocity [55], which may decrease slightly at MA, with dramatic decreases occurring at older ages [56]. Therefore, both responses should be studied to better understand age-related changes in postural muscle responses.

The brief EMG-BF neuromuscular training significantly improved PL preparation for landing only at MA. One possible explanation could be related to the principle of diminishing returns, which gives subjects with lower performance a greater chance to adapt and improve after training [57, 58]. Thus, MA adults who showed a lower PL anticipatory response responded to neuromuscular training, whereas young adults did not. In addition, our results showed no muscle fatigue after training in either group. These promising results should encourage further studies to investigate the effect of EMG-BF neuromuscular training on ankle muscle control. Such studies should examine different age groups with longer follow-up periods.

The present study had several limitations; therefore, our results should be interpreted with caution. The comparison of age performance measures can be influenced by many factors. Although we attempted to monitor the most important factors affecting aging, many other aspects were not monitored and may influence the results, such as proprioceptive sense and force generation ability. In addition, when recruiting subjects for the study, we specified that it should include a jump from a height of 25 cm and stabilization on one leg. This may have introduced a bias because MA adults who believed they could not perform this task or were afraid of it were not recruited. Other limitations relate to the fact that we focused only on the PL muscle, although there are several other ankle muscles that are important for maintaining stability [15], and the fact that we tested anticipatory activity in preparation for landing and did not assess the compensatory response after landing, which could also reveal differences between age groups. Therefore, further studies that will include a larger sample, and examine additional ankle muscles and compensatory responses are warranted. Finally, the study included only one day of testing, which may not be sufficient to ensure the consistency of the results. Nevertheless, we do not believe that these limitations had a major impact on our results. Therefore, our results should encourage further research on functional performance at MA. Such studies could promote appropriate interventions that might prevent further deterioration in third age.

## Conclusions

Automatic anticipatory peroneal responses in preparation for landing after a jump decreased at MA, whereas reflexive postural responses, after an unexpected inversion perturbation, appear to be intact in this age group. Furthermore, PL neuromuscular training with EMG-BF showed an immediate positive effect on postural response at MA. A better understanding of functional performance at MA would allow the development of specific interventions to ensure better postural control during aging.

## Supplementary Information

**Additional file 1: Appendix I.** Maximal voluntary contraction. **Appendix II.** Expected and unexpected fall measurement tools. **Appendix III.** The monitor representation of neuromuscular training. **Appendix IV.** The calculation of PL activity in preparation for landing.

## Data Availability

The data used in this manuscript are available from the corresponding author upon written request.

## Abbreviations

ANOVA: Analysis of Variance
EMG: Electromyography
EMG BF: Electromyography biofeedback
GRF: Ground reaction force
HHD: Handheld dynamometer
IQR: Interquartile range
IPAQ: International Physical Activity Questionnaire
IMU: Inertial motion unit
MA: Middle-aged
MANOVA: Multivariate analysis of Variance
MDF: Median frequency
MVC: Maximal voluntary contraction
PL: Peroneus longus
RMS: Root mean square
SD: Standard deviation
SLDJ: Single-leg drop-jump
SENIAM: Non-Invasive Assessment of Muscles
