# Supplementary material for: Peroneal muscle response to single-leg drop-jump and unexpected leg-drop in young and middle-aged adults before and after one session of neuromuscular training
Source: Eur Rev Aging Phys Act. 2023 Jun 17;20:11. doi: 10.1186/s11556-023-00321-8 (PMC10276378; doi:10.1186/s11556-023-00321-8)
Supplement: Supplementary file 1 — Additional file 1: Appendix I. Maximal voluntary contraction. Appendix II. Expected and unexpected fall measurement tools. Appendix III. The monitor representation of neuromuscular training. Appendix IV. The calculation of PL activity in preparation for landing. [file 11556_2023_321_MOESM1_ESM.docx]

***Appendix I:*** Maximal voluntary contraction


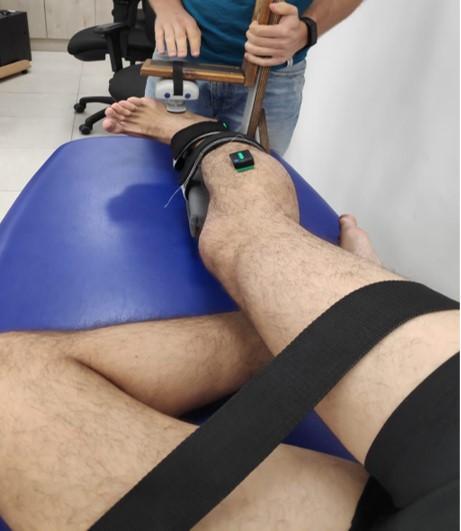


Hand-held dynamometer

Custom device

Subject positioned side-lying, on an adjustable treatment bed. Hand-held dynamometer, attached to the custom device, was placed on the subject's foot. The examiner stabilized the device during the measurement

**Appendix II:** Expected and unexpected fall measurement tools


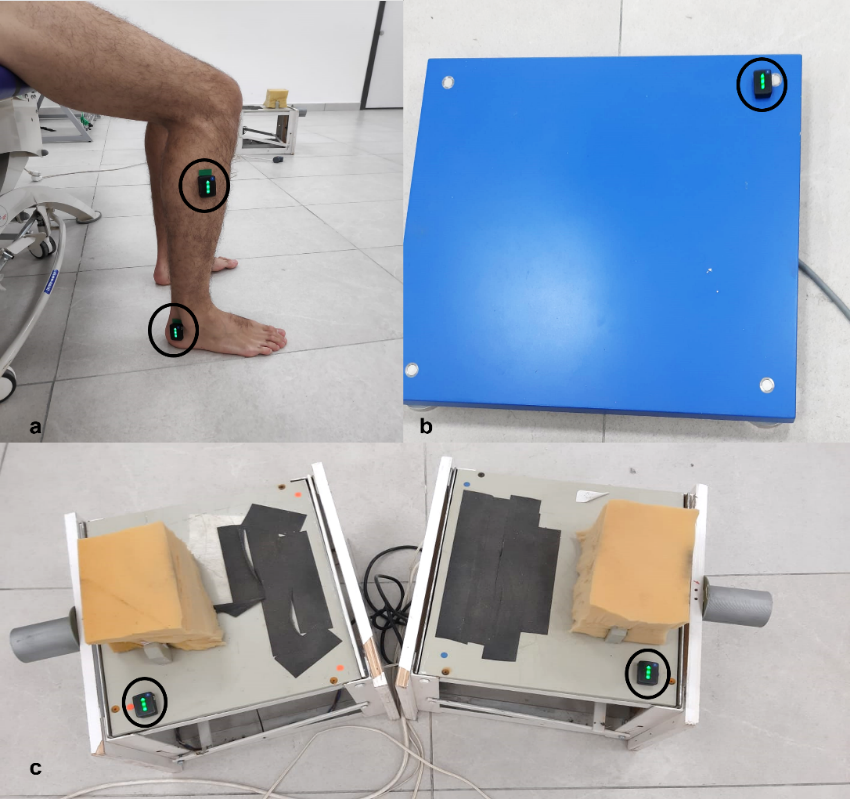


**(a)** Delsys sensors placement on subject's muscle ("PL EMG") and on subject's heel ("IMU heel"). **(b)** A 50⨯60⨯5 cm force plate (Kistler, Switzerland) and the Delsys sensor attached to the force plate ("IMU force plate"). **(c)** Delsys sensors placement on the right and left tilt platforms ("IMU rplatform and lplatform", respectively)

**Appendix III:** The monitor representation of neuromuscular training


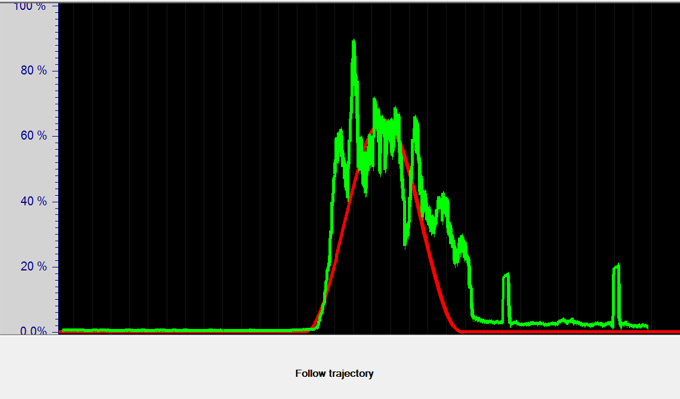


The red line represents the followed trajectory by the subjects. The green line represents muscle activity. Y axis represents percentages of muscle activity compared to maximal open kinematic chain contraction.

***Appendix IV:*** The calculation of PL activity in preparation for landing

To calculate PL activity in preparation for landing (% of flight time), we used the ACC signals from the "IMU heel" (H_ACC XYZ), the "IMU force plate" (FP _ACC_Z), and the EMG signal from the "PL EMG". First, missing values were removed, then, the landing time ($T_{L}$) was defined as the subject's first contact with the force plate after the jump, recorded from the FP _ACC_Z. The H_ACC and EMG signals were set to the same time points ($T_{L}$). The next step was to determine the onset of the jump ($O_{J}$) by forming a signal from H_ACC_XYZ, according to the formula:

${Acc}_{total}\sqrt{\left( ={H\_Acc}_{x} \right)^{2}+ \left( {H\_Acc}_{y} \right)^{2}+ \left( H\_{Acc}_{z} \right)^{2}}$ .

We used the "ischange" function to find changes in the signal (MaxNumChanges=3). The last change point represents the onset of the jump ($J_{O}$). The EMG signal was adjusted to start at the same time. The flight time ($T_{f}$) was defined as:

$$T_{f}=T_{L}-J_{O}$$

To determine PL preparation time ($P_{O}$) for landing, we used the EMG signal from the "PL EMG". We used a fourth-order Butter filter and frequency limits up to [20,400] divided by the EMG sampling rate. The filtered signal was smoothed using the "envelope" function, window 20 samples, method "rms". Changes in the smoothed signal were determined with the "ischange" function (MaxNumChanges=3). The last change point was marked as $P_{O}$ and the segment between this point and $T_{L}$ was the total preparation time ($T_{P}$). The $T_{P}$ was defined as:

$$T_{P}=T_{L}-P_{O}$$

The PL preparation percentage ($D_{PP}$) was defined by the following formula:

$$D_{PP}=\frac{T_{P}}{T_{f}}\cdot100\%$$
